# A 1000 fps High-Dynamic-Range Global Shutter CMOS Image Sensor with Full Thermometer Code Current-Steering Ramp

**DOI:** 10.3390/s25144483

**Published:** 2025-07-18

**Authors:** Liqiang Han, Ganlin Cheng, Xu Zhang, Gengyun Wang, Weijun Pan, Yao Yao, Guihai Yu, Ruimeng Zhang, Shuaichen Mu, Songbo Wu, Hongbo Bu, Liqun Dai, Ben Fan, Dan Wang, Wei Fan, Ruiming Chen

**Affiliations:** Beijing Institute of Space Mechanics and Electricity, Beijing 100094, China

**Keywords:** CMOS image sensor, global shutter pixel, current-steering DAC, readout noise

## Abstract

We present a 1024 × 512, 1000 fps, high-dynamic-range global shutter CMOS image sensor. The pixel is based on a voltage domain global shutter architecture, featuring a pitch of 24 μm × 24 μm. Both high-gain and low-gain signals can be captured within a single frame. The combined dynamic range is 95 dB, and the full well capacity is 620 ke-. In this paper, we analyze the pixel noise performance as well as the non-linearity and image lag that arise from parasitic capacitance in the pixel. The ramp generator is based on a 12-bit full thermometer code current-steering DAC with high driving capability. We discuss the design considerations for the ramp generator, particularly addressing the phenomenon of non-linear response. Finally, the comparator design and the column readout noise are analyzed.

## 1. Introduction

We present a CMOS image sensor (CIS) with a 24 μm × 24 μm global shutter pixel, suitable for scientific, industrial, and medical applications [1,2]. There are three types of global shutter (GS) pixel: a charge domain GS pixel, a voltage domain GS pixel and a pixel with pixel-parallel ADC [3,4]. The voltage domain GS pixel is particularly advantageous for large pixel designs or processes involving high-density metal capacitors [5], as it offers a larger full well capacity due to the independence of the voltage storage node from the pinning voltage. The 8T and 10T architectures represent two classical configurations for voltage domain GS pixels [6,7]. The correlated double sampling (CDS) operation is performed within the readout circuit. This paper analyzes both the noise performance of the voltage domain GS pixel and the requirements for the readout circuit. Additionally, we extend the dynamic range by employing four storage capacitors within each pixel, enabling simultaneous capture of high-gain and low-gain signals in a single frame.

Single-slope ADC (SS-ADC) is widely used in CISs [8]. In a CIS, the SS-ADC mainly consists of a ramp generator, a comparator and a counter. There are several architectures for the ramp design, including column level, chip level, and multi-column shared ramp generator. With a chip-level ramp generator, the SS-ADC can achieve better uniformity compared to SAR ADCs and cyclic ADCs [9]. The column level and multi-column shared ramps are typically based on current integration architecture, which suffers from random walk noise and kick-back noise [10,11]. For the design of large array CISs, the length of the ramp bus may extend several centimeters from one side of the array to the other. A distributed multiple ramp signal generator based on current integration architecture demonstrates good uniformity [12,13]. To achieve low-noise performance in such designs, a current-steering (CS) DAC can be employed as the ramp generator [14]. The heavy capacitive load presented by the column array effectively acts as a low-pass filter for the CS-DAC ramp, thus, its noise contribution can be considered negligible when compared to that from pixels and comparators. In conventional CS-DACs, current source arrays are generally organized using a segmented architecture that consists of a binary-coded least significant bit (LSB) array and a thermometer-coded most significant bit (MSB) array. However, in CIS applications where DAC functions as a ramp generator, sequential control over each current source within the array is necessary. This paper presents an innovative 12-bit full thermometer code current-steering DAC utilizing shift registers for digital control.

The comparator is a crucial component in SS-ADCs. Both dynamic comparators [14,15] and static comparators [4,11] can be implemented in a CIS. The comparator has two inputs: one is the output from the pixel or the programmable gain amplifier (PGA), while the other is the ramp signal. In the case of a static comparator, its first stage consists of a transconductance amplifier with ramp response, which can be modeled as a low-pass system [16]. This paper presents a comparator that incorporates a current compensation block. Additionally, we analyze the noise from the pixel source follower, column current source, column ramp buffer and the comparator itself.

Finally, the performance characteristics of this CIS are presented. It is fabricated in a standard 110-nm backside illumination (BSI) CIS process, and the array format is 1024 × 512. The frame rate achieves 1000 fps in single gain mode and 500 fps in dual gain mode. The combined dynamic range is measured at 95 dB.

## 2. CMOS Image Sensor Design

### 2.1. Global Shutter Pixel Design

#### 2.1.1. Basic Voltage Domain Global Shutter Pixel

In this design, the voltage domain GS pixel is used for a specific application. Metal capacitors can be employed for the storage node while ensuring a large full well capacity (FWC). The 8T and 10T pixels represent two classical configurations of voltage domain GS pixels. The CDS operation is performed within the column readout circuit.

**Figure 1:**
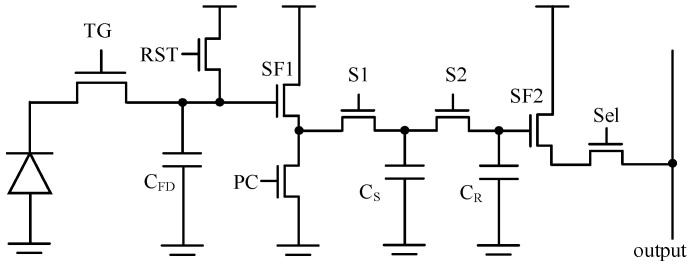
8T global shutter pixel.

**Figure 2:**
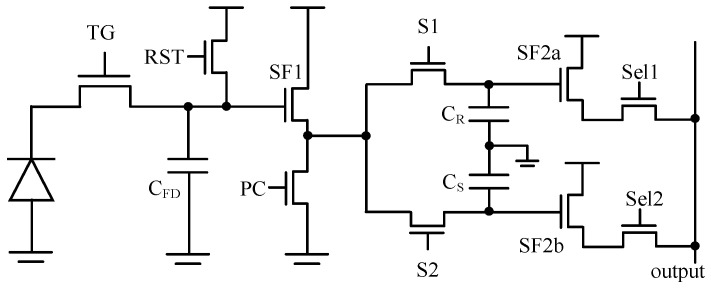
10T global shutter pixel.

**Figure 3:**
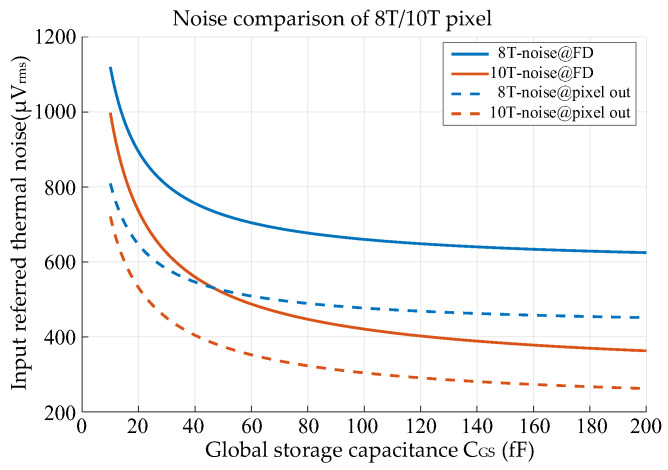
Input-referred noise comparison of 8T/10T pixel at *T* = 300 K, *gm_SF_*/*gm_PC_* = 0.4, *γ* = 2/3, *G_1_* = *G_2_* = 0.85, *V_n-col_* = 150 uV_rms_.

Figure 1 and Figure 2 show the basic 8T pixel and 10T pixel, respectively. The difference is the signal storage chain within each pixel. In the case of the 8T pixel, both the reset signal and light signal are read out through the same source follower (SF), which leads to charge-sharing between *C_S_* and *C_R_*. The input-referred thermal noise for both 8T and 10T pixels after CDS can be expressed as:

(1) $$V_{n-8T-FD}^{2}=2\times \left[\frac{kT\gamma }{G_{1}C_{GS}}\left(1+\frac{gm_{PC}}{gm_{SF1}}\right)+\frac{1}{0.5^{2}G_{1}^{2}G_{2}^{2}}V_{\mathrm{n-col}}^{2}\right]$$

(2) $$V_{n-10T-FD}^{2}=2\times \left[\frac{kT\gamma }{G_{1}C_{GS}}\left(1+\frac{gm_{PC}}{gm_{SF1}}\right)+\frac{1}{G_{1}^{2}G_{2}^{2}}V_{\mathrm{n-col}}^{2}\right]$$

where *k* is the Boltzmann constant, *T* is the absolute temperature, *γ* is the noise excess factor, *V_n-col_* is the thermal noise at the pixel output node originating from the SF2 to ADC chain, and *G_1_* and *G_2_* are the gains of SF1 and SF2, respectively. Additionally, *C_R_* and *C_S_* are the sampling capacitors in the pixel, both of which are equivalent to *C_GS_* in this discussion. The factor 2 represents CDS operation. The factor 0.5 in the second term represents the charge-sharing phenomenon within an 8T pixel. From Equations (1) and (2), we know that transconductance and capacitance are important for the voltage domain GS pixel design. Figure 3 shows the comparison results of thermal noise. Under the same column readout conditions, the noise performance of the 10T pixel is always better, especially for GS pixels with larger *C_GS_*. In other words, the 10T pixel relaxes the requirements on the column readout.

#### 2.1.2. High-Dynamic-Range 10T Pixel Design

**Figure 4:**
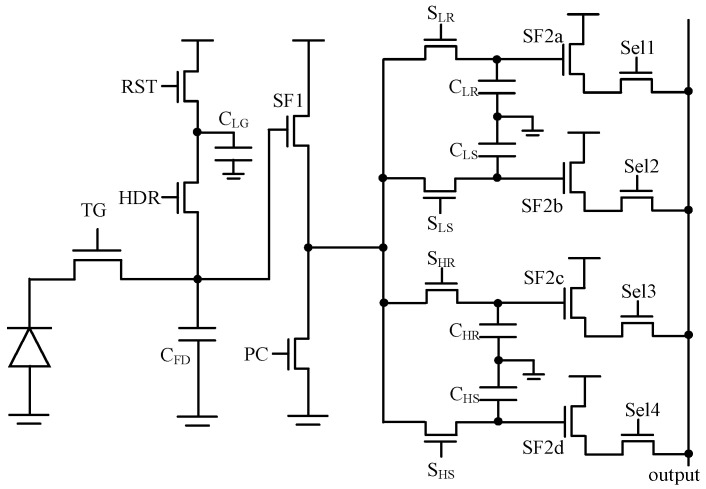
10T + HDR global shutter pixel. (TG, Transfer Gate; RST, reset transistor; HDR, high dynamic range switch; Sel, row select transistor).

Figure 4 shows the proposed high-dynamic-range global shutter pixel. There are two separate channels, enabling the capture of high conversion gain (HCG) and low conversion gain (LCG) signals within a single frame. S_LR_ + *C_LR_*, S_LS_ + *C_LS_*, S_HR_ + *C_HR_*_,_ and S_HS_ + *C_HS_* correspond to the LCG reset signal *V_LR_*, LCG light signal *V_LS_*, HCG reset signal *V_HR_*, and HCG light signal *V_HS_*, respectively. In this design, *C_LR_* = *C_LS_* = *C_HR_* = *C_HS_* = 120 fF, *C_FD_* = 4 fF, *C_LG_* = 60 fF, *gm_SF1_*/*id* = 15, and *gm_PC_*/*id* = 6. The estimated input-referred thermal noise at the FD node (*V_n-col_* = 0) is approximately 275 μV_rms_.

**Figure 5:**
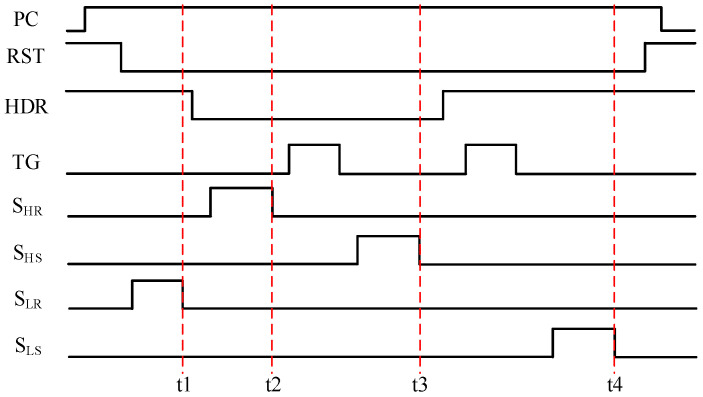
Timing diagram for the global charge transfer phase for a 10T + HDR global shutter pixel.

The parasitic capacitances in the proposed GS pixel affect both linearity and image lag performance. Figure 5 shows the timing diagram for the global charge transfer phase. Initially, *V_LR_* is stored on *C_LR_*_,_ and the voltage signal is locked at time t1. This indicates that *C_LR_* should be isolated from *C_HR_*_,_ *C_HS_* and *C_LS_* when *S_HR_*, *S_HS_* and *S_LS_* are set to high. For example, assuming that there is parasitic capacitance *C_LR-HS_* between *C_LR_* and *C_HS_*, the variation of *V_LR_* after the completion of *V_LS_* sampling can be expressed as:

(3) $$V_{LR}\left(\mathrm{frameN}.t3\right)-V_{LR}\left(\mathrm{frameN}.t1\right)=\frac{C_{LR-HS}}{C_{LR}}\left[V_{HS}\left(\mathrm{frameN}.t3\right)-V_{HS}\left(\mathrm{frameN}.t1\right)\right]$$

where frameN.t3 and frameN.t1 mean the voltage signal at times t3 and t1 within frame N, respectively.

(1) Non-linearity: When the in-pixel storage capacitors are reset for each frame, parasitic capacitance can significantly impact the linearity of the LCG signal, e.g., *C_LR-HS_* = 0.5 fF, *C_LR_* = 100 fF, V_HS_ variation from t1 to t3 is 1 V, and the result of V_LR_ variation calculated using Equation (3) is 5 mV. Given that the ratio of HCG to LCG is 15, this will affect the linearity of LCG’s response within the range of 0% to 15%. In contrast, within the range of 15% to 100% of LCG, HCG remains saturated, and a fixed offset will be observed in LCG’s response. Note that the parasitic capacitances *C_HR-HS_* and *C_LR-LS_* between the capacitors belonging to the same gain channel have an influence on the light response across the entire range.
(2) Image Lag: In the absence of a reset operation for the in-pixel storage capacitors following column readout, Equation (3) can be rewritten as:
  (4) $$V_{LR}\left(\mathrm{frameN}.t3\right)-V_{LR}\left(\mathrm{frameN}.t1\right)=\frac{C_{LR-HS}}{C_{LR}}\left[V_{HS}\left(\mathrm{frameN}.t3\right)-V_{HS}\left(\mathrm{frame}(\mathrm{N-1}).t3\right)\right]$$

The signal from the previous frame can influence the current frame in the presence of parasitic capacitance, e.g., *C_LR-HS_* = 0.1 fF, *C_LR_* = 100 fF, and assuming that the current frame is entirely dark while the HCG light signal from the previous frame is 1.2 V, the *V_LR_* variation calculated using Equation (4) is 1.2 mV.

For the same reason, C_HR_ should be isolated from both *C_HS_* and *C_LS_*, and *C_HS_* should be isolated from *C_LS_*_,_ as illustrated in the timing diagrams presented in Figure 5. Additionally, the parasitic capacitance between the source node of SF1 and the four storage capacitors also affects the signal accuracy. In conclusion, it is essential to minimize the parasitic capacitances among all five nodes, including the source node of SF1 and the four storage capacitors.

**Figure 6:**
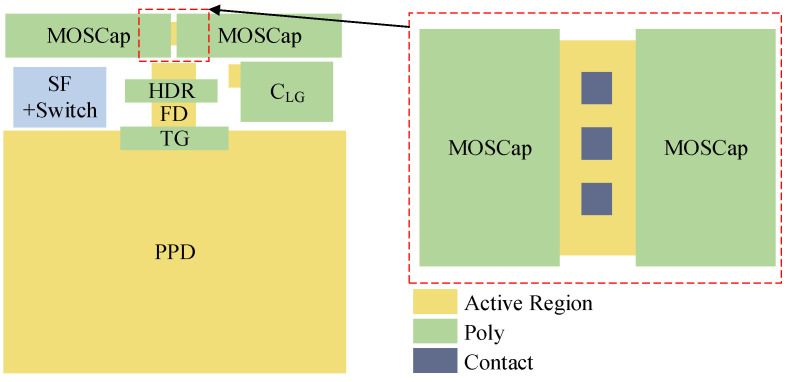
Pixel layout example with isolated MOS capacitors.

Figure 6 shows an example of the pixel layout. The pixel pitch is 24 μm and the fill factor is 70%. The four storage capacitors are utilized by two MOS capacitors and two MIM capacitors. The two MOS capacitors are isolated from each other by the contacts located between metal 1 and the active region. These contacts are connected to the ground and, thus, shield the electric field lines. In this design, the parasitic capacitances among the five nodes remain below 0.01 fF.

### 2.2. Readout Circuits Design

#### 2.2.1. Readout Architecture

The output swing of the proposed pixel is approximately 1.3 V, utilizing a design with a 1.4 V pinning voltage (Vpin) and 0.85~0.88 SF gain. As previously mentioned, the input-referred thermal noise of the GS pixel before the SF2 stage is 275 μV_rms_, which means the noise performance of the sensor is limited by the pixel if the ADC resolution ≥ 12 bits. The quantizing noise of a 12-bit ADC is 92 μV_rms_ within a range of 1.3 V, and the total noise for such an ADC generally remains below 1 LSB. In this design, GS pixel + column ADC architecture is used.

**Figure 7:**
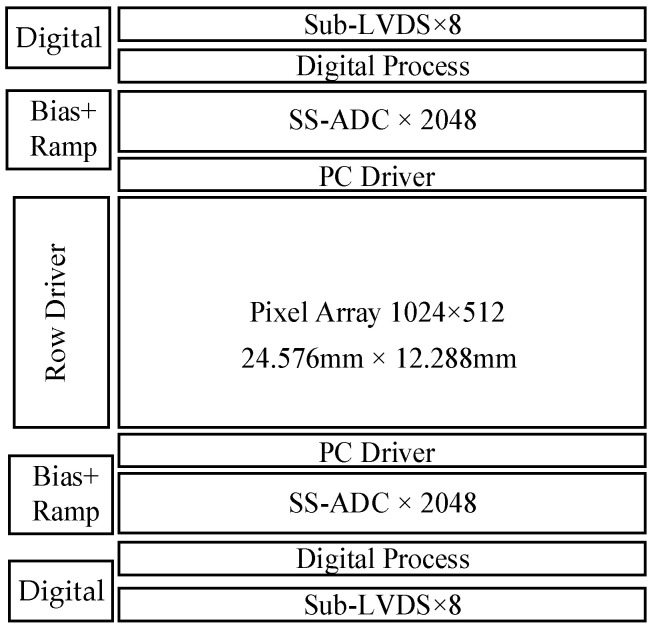
Architecture of the proposed CMOS image sensor.

The array format of this sensor is 1024 × 512, with a maximum frame rate of 1000 fps in single gain mode. The effective row time for one column is 1.9 μs. Figure 7 shows the architecture of the proposed CIS. Due to the 24 μm column width and the limitation of the 110 nm CMOS process, there are four SS-ADCs for each pixel column. The clock frequency for the counter operates at 500 MHz. To match the pixel output swing, the ADC input range is slightly higher than 1.3 V at × 1 gain.

#### 2.2.2. PC Driver Design

In the GS pixel, the pre-charge (PC) transistor is utilized for voltage sampling. The charging current is determined by both the capacitance of the storage node and the requirements for settling time. Typically, the current *I_PC_* ranges from several hundred nA to 1 μA. In this design, the current *I_PC_* is set at 1 μA, and the duration of the global charge transfer phase is 10 μs. Consequently, the total current across the entire pixel array during this phase approximates 524 mA. Therefore, it is important to consider the topology of the power supply for the voltage domain GS pixel array.

**Figure 8:**
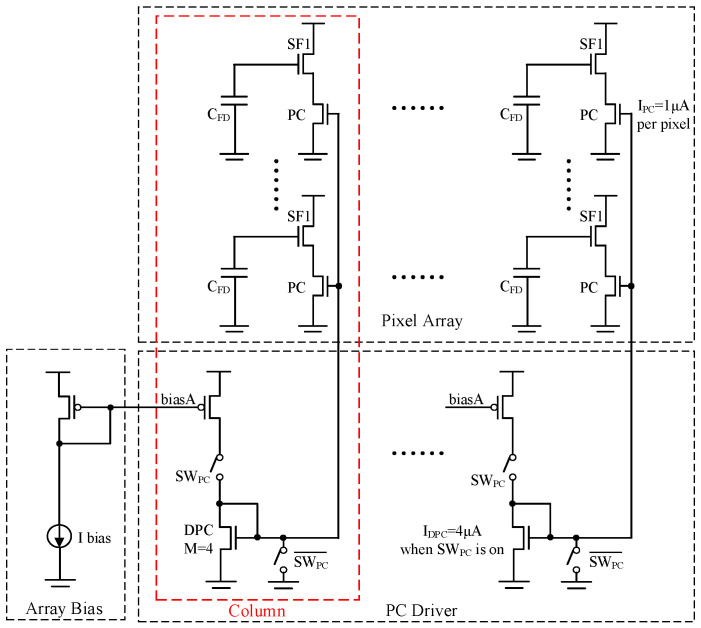
Architecture of the PC driver circuit.

Figure 8 shows the PC driver circuit, which is based on the current mirror circuit. The nMOS transistor DPC must be identical to the type of PC transistor used in the pixel, and thus the current *I_PC_* in a pixel can be exactly controlled. To ensure that the settling time of the column bus for the PC bias remains under 1 μs, the current of the PC driver per column *I_DPC_* is set to 4 × *I_PC_* in this design.

#### 2.2.3. Ramp Generator Architecture

The input range of the SS-ADC is directly determined by the ramp reference. There are several architectures for the ramp design, including column level, chip level and a multi-column shared ramp generator. A chip-level ramp generator is employed to achieve better uniformity.

**Figure 9:**
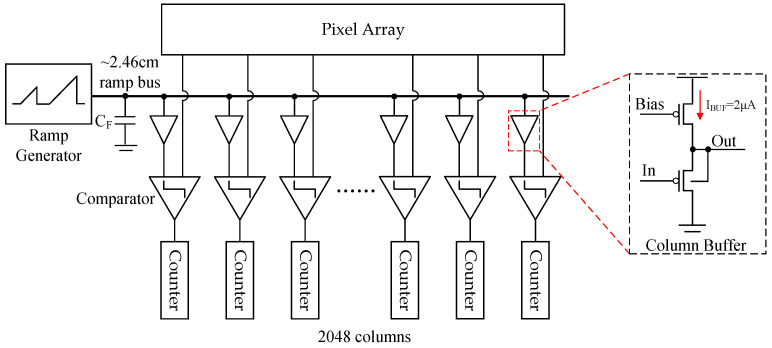
Architecture of the ramp generator and column load.

In this sensor, the length of the ramp bus is approximately 2.46 cm. The row time of the column ADC is 7.6 μs at 12-bit resolution operation. High driving capability is necessary for the ramp generator, which is utilized by a current steering DAC. Figure 9 shows the architecture of the ramp generator and column load. There is a pMOS SF buffer for each column between the ramp bus and the comparator. The column ramp buffer consumes 2 μA current. The noise originating from the column ramp buffer can be effectively filtered by the comparator, and it will be discussed in more detail later.

**Figure 10:**
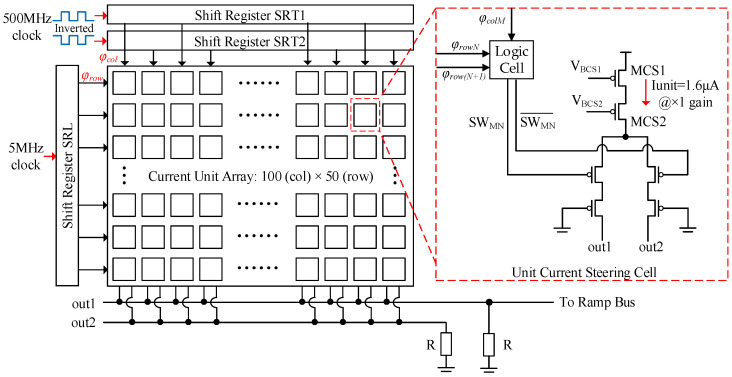
Architecture of the ramp generator.

**Figure 11:**
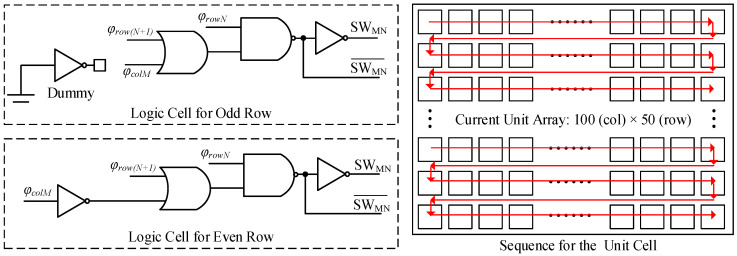
Logic in a unit current steering cell.

Different from a classical current steering DAC application, the DAC output in a CMOS image sensor is certainly a ramp signal. It means that the control sequence for each unit cell operates sequentially. So a full thermometer code current-steering DAC is employed in this design, which effectively eliminates output glitches. Figure 10 shows the architecture of the proposed ramp generator. It is basically a current steering cell array with 100 columns × 50 rows. The total array consists of 5000 cells and supports digital double sampling (DDS) operation at a resolution of 12-bit. A maximum of 904 (5000 − 4096) cells can be used for the ramp adjustment. To generate the thermometer code, three shift register blocks are employed. The column control signal *φ_col_* is derived from two identical shift registers, SRT1 and SRT2, by utilizing an inverted input clock operating at 500 MHz. The row control signal *φ_row_* is generated from register SRL with a 5-MHz input clock. The unit current steering cell (UCSC) consists of a current steering circuit and a logic cell. Figure 11 shows the logic cell in a UCSC. For odd rows, current flows to out1 when *φ_col_* = high, *φ_rowN_* = high, and *φ_row(N+1)_* = low. For even rows, the current flows to out1 when *φ_col_* = low, *φ_rowN_* = high, and *φ_row(N+1)_* = low. When *φ_row(N+1)_* = high, the current of the entire row *N* flows to out1.

The linearity of a CIS with pinned photodiode is usually limited by the pixel [17]. The non-linearity of a CIS is typically 1% ± 0.5%, which is highly dependent on the design of the FD node and the source follower in the pixel. The measured non-linearity of the entire sensor is 1.09% at high gain and 0.78% at low gain, respectively. The geometry of the MCS1 in the UCSC and the overdrive voltage are optimized for the purpose of mismatch improvement. In this sensor, the geometry of MCS1 is W/L = 0.5 μm/1 μm, and the default current of the UCSC is 1.6 μA, which can be adjusted to achieve different analog gains. The simulated mismatches of the current for one UCSC and for an array of 4096 USCSs are 1.38% (1σ) and 0.0214% (1σ), respectively. In this sensor, the load resistor *R* is 200 Ω, and the voltage swing of 4096 steps is 1.31 V at × 1 gain. The area of the ramp generator is 570 μm (H) × 320 μm (V).

#### 2.2.4. Ramp Generator Analysis

**Figure 12:**
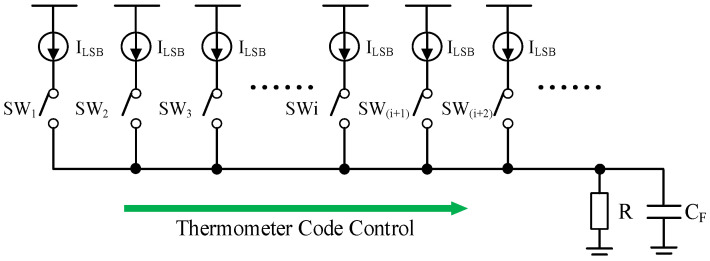
Simplified schematic diagram of the ramp.

The simplified schematic diagram of the proposed ramp is presented in Figure 12. The resistance *R* should match the requirements for the driving capability. The capacitance *C_F_* discussed in this chapter consists of both the parasitic capacitance and the load of the ramp. The effective resistance of the unit current source exceeds one million Ω at least, while the load resistance *R* is generally several hundred Ω. Consequently, the finite resistance of the current source is neglected in the following discussion.

**Figure 13:**
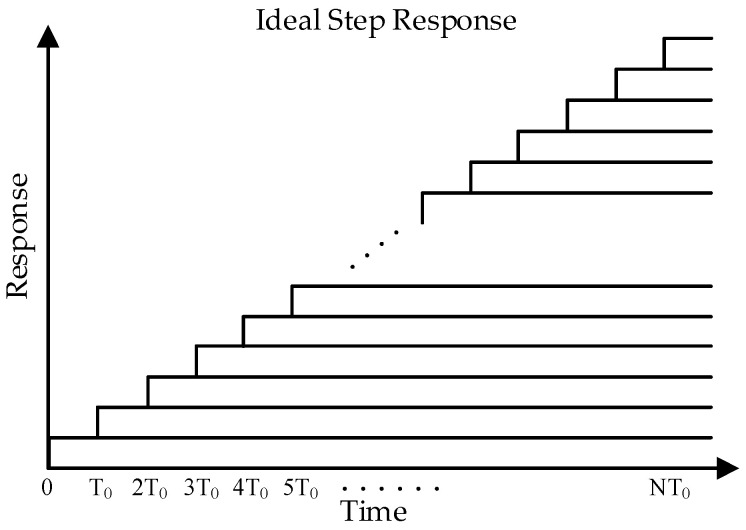
Summation of multiple unit step responses.

The step response of the 1 st LSB current source in time domain is given by:

(5) $$V_{LSB-1}(t)=I_{LSB}R\times \left[1-\exp(-\frac{t}{\tau })\right]\times u(t)$$

where *τ* = *RC_F_* is the time constant. The step response of the *i*-th LSB current source with a time shift (*i* − 1)*T_0_* can be expressed as:

(6) $$V_{LSB-i}(t)=I_{LSB}R\times \left[1-\exp(-\frac{t-(i-1)T_{0}}{\tau })\right]\times u\left[t-(i-1)T_{0}\right]$$

where *T_0_* is the time for one step. As shown in Figure 13, the ideal response of the ramp can be considered as the summation of multiple unit step responses with different time shifts. This relationship can be expressed as:

(7) $$V_{\mathrm{total}}(t)=\sum_{i=1}^{N}I_{LSB}R\times \left[1-\exp(-\frac{t-(i-1)T_{0}}{\tau })\right]\times u\left[t-(i-1)T_{0}\right]$$

**Figure 14:**
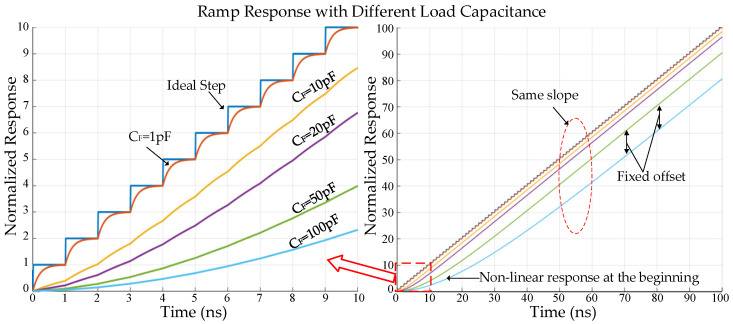
Ramp response with different *C_F_* (*R* = 200 Ω, *T_0_* = 1 ns).

Figure 14 shows an example of the ramp response curves with different *C_F_* values, where *T_0_* = 1 ns and *R* = 200 Ω. Under the condition that *τ* << *T_0_*, e.g., *τ* = 0.2 ns (*C_F_* = 1 pF, *R* = 200 Ω), distinct steps can be observed. Under the condition that *τ* > *T_0_*, the step disappears, and a smooth curve is obtained. A non-linear response phenomenon is observed at the very beginning of the curve. If the time is long enough, e.g., several tens of nanoseconds, the slopes of the curves with different *τ* will converge to the same value but maintain a fixed offset from one another. In this design, the parasitic capacitance of the ramp output node is approximately 15 pF, which means that the ramp output can be directly connected to the column array without a real capacitor between the two blocks.

Analyzing the response between t = (*N* − 1)*T_0_* and t = *NT_0_*, we obtain:

(8) $${\left.V_{\mathrm{total}}(t)\right|}_{(N-1)T_{0}\leq t<NT_{0}}=\left\{I_{LSB}R\times \sum_{i=1}^{N}\left[1-\exp(-\frac{t-(i-1)T_{0}}{\tau })\right]\right\}\times u\left[t-(N-1)T_{0}\right]$$

(9) $${\left.V_{\mathrm{total}}(t)\right|}_{(N-1)T_{0}\leq t<NT_{0}}=\left\{I_{LSB}R\left[N-\exp\left(-\frac{t-(N-1)T_{0}}{\tau }\right)\right]\times \frac{\exp\left(-NT_{0}/\tau \right)-1}{\exp\left(-T_{0}/\tau \right)-1}\right\}\times u\left[t-(N-1)T_{0}\right]$$

The output increment between t = (*N* − 1)*T_0_* and t = *NT_0_* is given by:

(10) $$\Delta V_{\mathrm{step}}=V_{\mathrm{total}}\left[(N-1)T_{0}+t\right]-V_{\mathrm{total}}\left[(N-1)T_{0}\right]$$

(11) $$\left\{\begin{array}{l} \Delta V_{\mathrm{step}}=I_{LSB}R\times G_{N}\times R_{\mathrm{step}}\\ G_{N}=1-\exp\left(-NT_{0}/\tau \right)\\ R_{\mathrm{step}}=\left[1-\exp\left(-t/\tau \right)\right]/\left[1-\exp\left(-T_{0}/\tau \right)\right] \end{array}\right.$$

**Figure 15:**
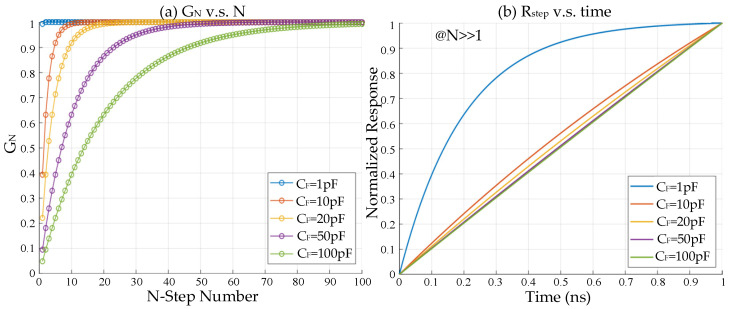
(**a**) *Gain* vs. *N*; (**b**) ramp response of unit step (*R* = 200 Ω, *T_0_* = 1 ns).

The gain *G_N_* is determined by the settling time of the ramp, with a range from 0 to 1. *R_step_* represents the ramp response of a unit step under the condition that *N* >> 1. Figure 15a shows the relationship between *G_N_* and *N*. Assuming that the acceptable gain error is <1%:

(12) $$Gain\_Error=\exp\left(-NT_{0}/\tau \right)<1\%$$

(13) $$N_{min}>\frac{\tau }{T_{0}}\ln\left(\frac{1}{1\%}\right)$$

Under the condition that *R* = 200 Ω and *T_0_* = 1 ns, the minimum number of steps *N_min_* to achieve the target gain 0.99 when *C_F_* = 1 pF, *C_F_* = 10 pF, *C_F_* = 20 pF, *C_F_* = 50 pF, and *C_F_* = 100 pF are 1, 10, 19, 47, and 93, respectively. Figure 15b shows the relationship between *R_step_* and time. The response curve approximates a straight line when *τ* = *RC_F_* is sufficiently large. The maximum difference between *R_step_* and the ideal ramp when *C_F_* = 15 pF is 0.04 LSB, which is given by:

(14) $$\Delta V_{\mathrm{error-MAX}}=\max\left\{R_{\mathrm{step}}-\frac{t}{T_{0}}\right\}$$

The 0.04 LSB difference can be neglected even without an additional capacitive load. In this design, the output of the ramp is connected to the column array, which can be considered as an RC network. The simulated time delay for the ramp response from the first column to the 2048th column is 22 ns. The influence of the delay could be easily compensated through DDS operation and 904 redundant UCSCs.

According to Equations (11)–(14), the load resistor R and the clock frequency are important for implementing current-steering ramps in a CIS. For CISs operating at relatively low frame rates, the resolution of the ramp could be increased by using a real capacitor between the ramp and column array, e.g., a 14-bit ramp can be achieved from a 12-bit current-steering ramp when coupled with a suitable filtering capacitor. For CISs designed for high frame rates, utilizing a smaller resistance R is advantageous as it reduces the time constant, e.g., *R* = 100 Ω. Due to the high driving capability of the ramp and the isolation provided by the column ramp buffer, this architecture could be used in a stitched CIS, and the parasitic RC of the ramp bus must be carefully controlled.

#### 2.2.5. Comparator Design

**Figure 16:**
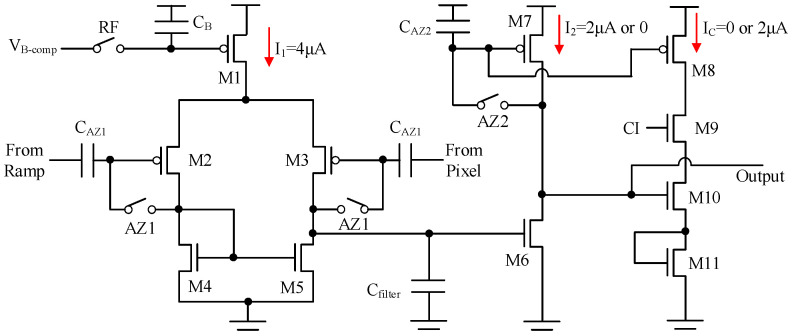
Architecture of the comparator with current compensation.

In this CIS, the output of the pixel is directly connected to the input of the comparator without a sample and hold circuit between them. Figure 16 shows the architecture of the comparator. It is based on a two-stage static comparator with a current compensation block. The first stage of the comparator consists of a transconductance amplifier (M1-M5), auto-zero switches AZ1 and capacitors *C_AZ1_*, a decoupling capacitor *C_B_*, and a refresh switch RF. The second stage consists of M6-M11, auto-zero switch AZ2 and capacitor C_AZ2_. In this design, *C_AZ1_* = 400 fF, *C_AZ2_* = 600 fF, *C_B_* = 600 fF, and dummy switches are employed.

**Figure 17:**
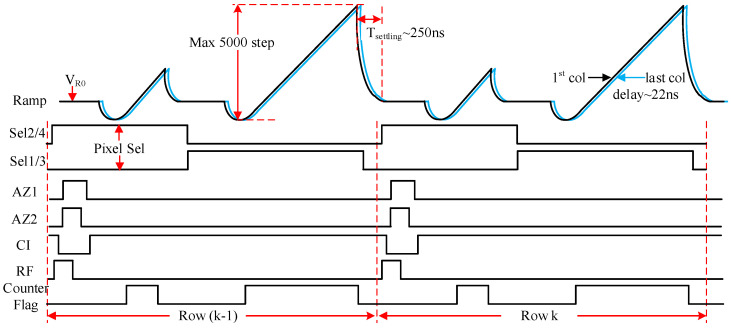
Timing diagram for the column readout.

Figure 17 shows the timing diagram for the column readout. At the beginning of each cycle, the comparator is reset by the auto-zero switches AZ1 and AZ2 to improve offset and mismatch. Additionally, the decoupling capacitor *C_B_* is reset to optimize the row-wise noise. The effective input capacitance of the comparator when AZ1 is on is 400 fF (*C_AZ1_*), while it drops to 20 fF when AZ1 is off. Both AZ1 and AZ2 turn on after the full-range ramp resets to *V_R0_*. Due to the voltage domain GS pixel architecture, light signals can be read out first, which corresponds to the small ramp. Between the two ramps, the reset signal is selected for output transmission. It should be noted that the pixel timing and the operational points must be optimized in order to prevent unintended activation of switch AZ1. CI is the timing control for current compensation. When the counter flag is high, the summation of *I_2_* and *I_C_* remains nearly constant, thereby improving IR-drop performance across the entire array.

The noise performance can be improved as follows:

(1) To improve low-frequency noise and avoid column flicker noise originating from the input transistors of the column readout, pMOS input transistors M2 and M3 are employed. The area of the input transistor is 10 μm^2^ for the purpose of low-frequency noise improvement.
(2) The first stage of the comparator is actually an operational transconductance amplifier. The transfer function for a step ramp t × u (t) is 1/s^2^. The response in the time domain can be written as [8]:

(15) $$V_{\mathrm{out-1st}}=K_{ramp}gm_{1st}R_{1st}\cdot \left\{t-\tau_{1st}\left[1-\exp\left(-\frac{t}{\tau_{1st}}\right)\right]\right\}\times u(t)$$

where *K_ramp_* is the slope of the ramp, *gm_1st_* is the transconductance of the first stage, *R_1st_* is the output impedance of the first stage, and *τ_1st_* is the time constant. The average crossing time *T_i_* for the entire comparator is shorter than *τ_1st_*. Consequently, Equation (15) can be simplified by taking a second-order Taylor series expansion:

(16) $$V_{\mathrm{out-1st}}=K_{ramp}gm_{1st}R_{1st}\cdot \left\{t-\tau_{1st}\left[\frac{t}{\tau_{1st}}-\frac{1}{2!}{\left(\frac{t}{\tau_{1st}}\right)}^{2}\ldots \right]\right\}\times u(t)\;t\ll \tau_{1st}$$

(17) $$V_{\mathrm{out-1st}}\approx \frac{K_{ramp}gm_{1st}t^{2}}{2C_{filter}}\times u(t)\;t\ll \tau_{1st}$$

Assuming the threshold voltage for the second stage transition is *Vth-2nd* and the gain of the second stage is sufficiently large. The time average crossing time *T_i_* can be expressed as:

(18) $$T_{i}\approx \sqrt{\frac{2C_{filter}V_{\mathrm{th-2nd}}}{K_{ramp}gm_{1st}}}\;T_{i}\ll \tau_{1st}$$

For the first stage, the noise bandwidth of the input-referred noise is similar to the usual one-pole system, and the effective noise bandwidth can be written as [16]:

(19) $$NBW=\frac{1}{2T_{i}}\;T_{i}\ll \tau_{1st}$$

By substituting Equation (18) into (19), we obtain:

(20) $$NBW\approx \sqrt{\frac{K_{ramp}gm_{1st}}{8C_{filter}V_{\mathrm{th-2nd}}}}\;T_{i}\ll \tau_{1st}$$

Actually, the noise originated from pixel SF2, the column current source and the transconductance amplifier can be effectively filtered within the time window *T_i_* by the sinc-type filter. Thus, the thermal noise at the pixel output node before DDS can be estimated as:

(21) $$V_{\mathrm{n-}pixe\mathrm{l-}out}=\sqrt{NBW\times \frac{\pi }{2}\times 4kT\gamma \left[\frac{G_{2}^{2}\left(gm_{SF2}+gm_{CS}\right)}{gm_{SF2}^{2}}+\frac{gm_{1st}+gm_{1st-L}}{gm_{1st}^{2}}+\frac{gm_{buf-in}+gm_{buf-L}}{gm_{buf-in}^{2}}\right]}$$

where *γ* is the noise excess factor, *gm_SF2_*, *gm_CS_*, *gm_1st-L_*, *gm_buf-in_* and *gm_buf-L_* are the transconductance of pixel SF2, column current source, comparator load transistors of the first stage (M4/M5) and column ramp buffer transistors, respectively. The CDS/DDS operation will result in a doubling of the thermal noise power. An increased value of *gm_1st_* is beneficial for reducing noise from the comparator, however, it worsens the noise from SF2 and the column ramp buffer due to the increase in NBW. Usually, *gm_SF2_* is sufficiently large for fast settling. For low-noise applications, *gm_buf-in_* should be large enough, and this requirement may lead to higher power consumption.

**Figure 18:**
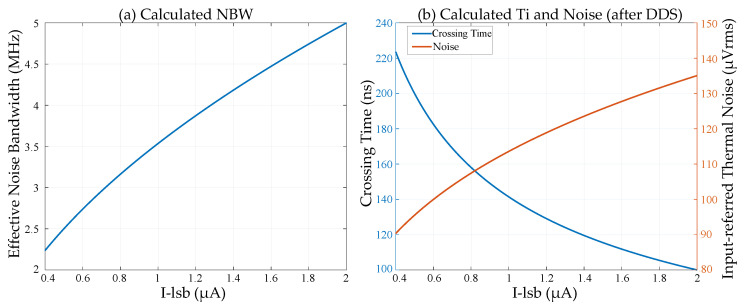
(**a**) Calculated NBW; (**b**) calculated *T_i_* and noise at 300 K (thermal noise after DDS).

Figure 18 shows the calculation results derived from Equations (18), (20) and (21). The parameters used in the calculations are as follows: *K_ramp_* = 0.32 V/μs at ×1 gain, *gm_1st_* = *gm_buf-in_* = 30 μS, *gm_SF2_* = 120 μS, *gm_CS_* = 50 μS, *gm_1st-L_* = 15 μS, *gm_buf-L_* = 12 μS, *R_1st_* = 14 MΩ, *G_2_* = 0.85, *Vth-2nd* = 0.4 V (low-VT nMOS), and *C_filter_* = 150 fF (including parasitic capacitance). The noise bandwidth decreases with the increase of the ramp slope, *K_ramp_*. The calculated thermal noise at ×1 gain (*I_LSB_* = 1.6 μA) and ×4 gain (*I_LSB_* = 0.4 μA) after DDS are 128 μV_rms_ and 90 μV_rms_, respectively.

**Figure 19:**
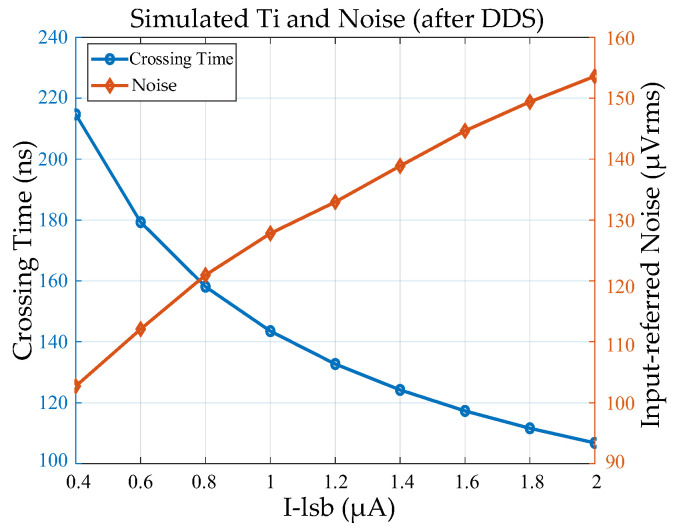
Simulation results: *T_i_* and noise at 300 K (including thermal and 1/f noise).

Figure 19 shows the circuit simulation results of the crossing time and the input-referred noise after DDS, which includes thermal noise and low-frequency noise originating from the comparator, pixel SF2, column current source and column ramp buffer. The interval *T_DDS_* between the two crossing points of DDS signals is 1.2 μs under dark conditions, which affects the low-frequency noise performance according to the CDS/DDS transfer function 2 × cos(2πf*T_DDS_*). The simulated input-referred noise at ×1 gain (*I_LSB_* = 1.6 μA) and ×4 gain (*I_LSB_* = 0.4 μA) after DDS are 145 μV_rms_ and 103 μV_rms_, respectively.

## 3. Results

**Figure 20:**
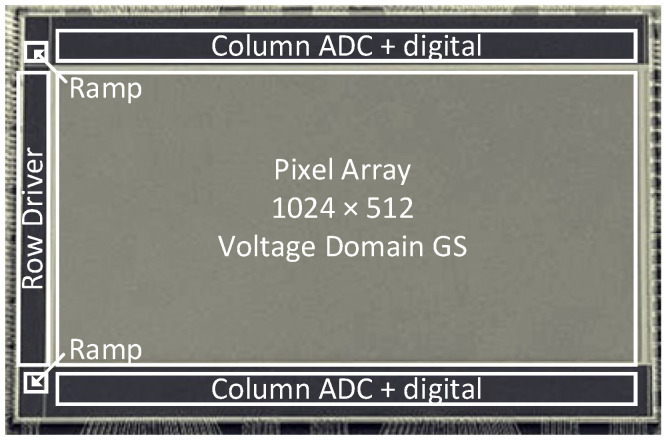
The photograph of the CMOS image sensor.

**Figure 21:**
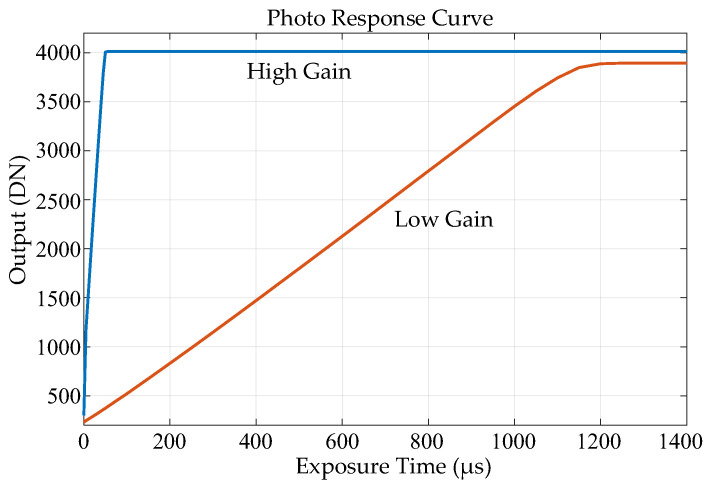
Photo response curves captured at dual gain mode. (Blue: high gain response; Red: low gain response).

**Figure 22:**
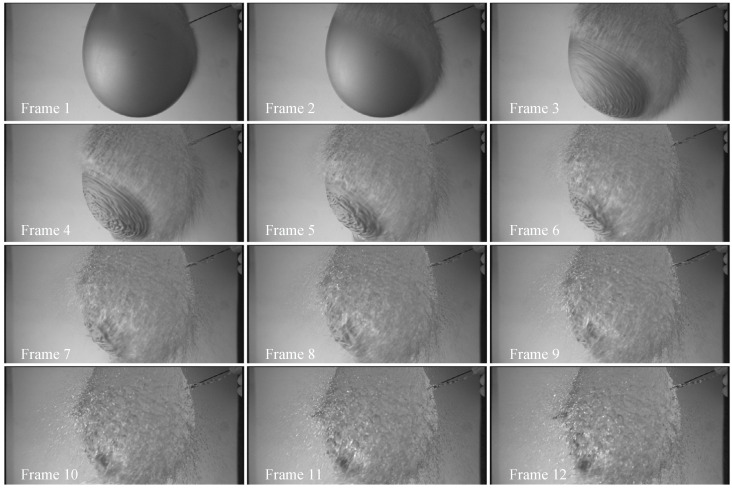
Twelve consecutive frames captured at 1000 fps: piercing a water balloon (raw image).

Our sensor was fabricated with a standard 110 nm backside illumination (BSI) CIS process. The pixel pitch was 24 μm, and the array format is 1024 × 512; Figure 20 shows a photograph of the sensor. Both HCG and LCG images can be captured within a single frame. Figure 21 shows the photo response curves captured in dual gain mode. In this sensor, the FWC of the HCG signal was limited by the FD node, with a maximum output of 4096 DN. The FWC of the LCG signal was limited by the photodiode, resulting in a maximum output that is slightly lower than 4096 DN. The sensitivity ratio between HCG and LCG was approximately 16:1. The pixel process was optimized, and the FWC for LCG was 620 ke-. The noise in HCG mode was 10e-_rms_ at ×1 ADC gain (*I_LSB_* = 1.6 μA), which corresponds to 320μV_rms_. The combined dynamic range was 95 dB, and the linear dynamic range was 71 dB. Figure 22 shows an example of high-frame-rate imaging: 12 consecutive frames captured at 1000 fps. See Table 1, Table 2 and Table 3.

## 4. Discussion

In this paper, we presented a 1024 × 512 global shutter CMOS image sensor. The pixel was based on the voltage domain global shutter architecture, and the pitch was 24 μm × 24 μm. The frame rate was 1000 fps in single-gain mode and 500 fps in dual-gain mode. The combined dynamic range was 95 dB, and the full well capacity was 620 ke-. We analyzed the pixel noise performance, the non-linearity, and the image lag that originated from the parasitic capacitance within the pixel. A 10T pixel with dual gain channels was implemented to relax the requirements for the readout circuit. The parasitic capacitances in the pixel were smaller than 0.01 fF to minimize lag and non-linearity.

The ramp generator was based on a 12-bit full thermometer code current-steering DAC with high driving capability. We analyzed the non-linear response phenomenon of the ramp. The length of the ramp bus was approximately 2.46 cm. Theoretically, this architecture can be used in a stitched CIS. A static comparator with a current compensation block was also discussed. The noise from the pixel source follower, column current source, column ramp buffer, and the comparator itself was analyzed, which can guide the design of low-noise readout.

## Figures and Tables

**Table 1:** Performance summary.

Parameter	Specification
Technology	110 nm BSI
Pixel Pitch	24 μm × 24 μm
Array Format	1024(H) × 512(V)
Pixel Type	voltage domain global shutter pixel
ADC Resolution	12-bit
Frame Rate	1000 fps at single gain; 500 fps at dual gain
Full Well Capacity	620 ke- at low gain; 37 ke- at high gain
Temporal Noise	10e-_rms_ at high gain and ×1 ADC gain
Image Lag	<0.08%
Dynamic Range	95 dB
PRNU	2.26% at high gain, 1.25% at low gain
FPN	0.74%
Dark Current	3200e-/s at 40 °C die temperature
Non-linearity (5%~90%)	1.09% at high gain; 0.78% at low gain
Quantum Efficiency	60% at 550 nm
Output Data	16 sub-LVDS at 500 MHz

**Table 2:** Power consumption measured at 1000 fps single gain mode under dark environment.

Module	Power Consumption
Digital + Counter	155 mW
Pixel Array + PC bias	86 mW
Column Readout (Analog)	168 mW
Ramp Generator	64 mW
Sub-LVDS	73 mW
Row Driver	11 mW
Total	557 mW

**Table 3:** Performance comparison.

Parameter	This Work	IISW 2015 [1]	IEEE-TED 2021 [2]	GSPRINT6502 [18]	CAE302 [19]
Technology	110 nm BSI	-	180 nm	-	-
Pixel Type	voltage domain global shutter	voltage domain global shutter	voltage domain global shutter + LOFIC	global shutter	global shutter
Pixel Pitch	24 μm × 24 μm	20 μm × 20 μm	22.4 μm × 22.4 μm	6.5 μm × 6.5 μm	15.5 μm × 15.5 μm
Array Format	1024 × 512	1024 × 1024	160 × 88	2048 × 1152	2048 × 256
Full Well Capacity	620 ke-	510 ke-	21.9 Me-	10.6 ke-	200 ke-
Temporal Noise	10e-_rms_	27e-_rms_	8.1e-_rms_	21.3e-_rms_	9e-_rms_
Dark Current	3200e-/s at 40 °C	6000e-/s at 25 °C	-	-	10 pA/cm^2^ at 25 °C
Frame Rate	1000 fps at single gain mode	-	450 fps	1498 fps	205 fps
ADC resolution	12-bit	analog output	analog output	10-bit	analog output

## Data Availability

The original contributions presented in this study are included in the article. Further inquiries can be directed to the corresponding author.
